# Systematic review and meta-analysis of the effects of exercise on depression in adolescents

**DOI:** 10.1186/s13034-022-00453-2

**Published:** 2022-02-28

**Authors:** Xiang Wang, Zhi-dong Cai, Wan-ting Jiang, Yan-yan Fang, Wen-xin Sun, Xing Wang

**Affiliations:** School of Physical Education and Training, Shanghai University of Sport, 650 Qingyuan Ring Road, Yangpu District, Shanghai, 200438 China

**Keywords:** Exercise, Adolescent, Depression, Depressive symptoms, Meta-analysis, Systematic review

## Abstract

**Background:**

Depression is widespread among adolescents and seriously endangers their quality of life and academic performance. Developing strategies for adolescent depression has important public health implications. No systematic review on the effectiveness of physical exercise for adolescents aged 12–18 years with depression or depressive symptoms has previously been conducted. This study aims to systematically evaluate the effect of physical exercise on adolescent depression in the hope of developing optimum physical exercise programs.

**Methods:**

Nine major databases at home and abroad were searched to retrieve randomized controlled trials (RCTs) on exercise interventions among adolescents with depression or depressive symptoms. The retrieval period started from the founding date of each database to May 1, 2021. The methodological quality of the included articles was evaluated using the modified PEDro scale. A meta-analysis, subgroup analysis, sensitivity analysis, and publication bias tests were then conducted.

**Results:**

Fifteen articles, involving 19 comparisons, with a sample size of 1331, were included. Physical exercise significantly reduced adolescent depression (standardized mean difference [SMD] = − 0.64, 95% CI − 0.89, − 0.39, p < 0.01), with a moderate effect size, in both adolescents with depression (SMD = -0.57, 95% CI − 0.90, − 0.23, p < 0.01) and adolescents with depressive symptoms (SMD = − 0.67, 95% CI − 1.00, − 0.33, p < 0.01). In subgroups of different depression categories (depression or depressive symptoms), aerobic exercise was the main form of exercise for the treatment of adolescents with depression. For adolescents with depression, interventions lasting 6 weeks, 30 min/time, and 4 times/week had optimum results. The effects of aerobic exercise and resistance + aerobic exercise in the subgroup of adolescents with depressive symptoms were significant, while the effect of physical and mental exercise (yoga) was not significant. For adolescents with depressive symptoms, aerobic exercise lasting 8 weeks, 75–120 min/time, and 3 times/week had optimum results. Physical exercise with moderate intensity is a better choice for adolescents with depression and depressive symptoms.

**Conclusions:**

Physical exercise has a positive effect on the improvement of depression in adolescents.

The protocol for this study was registered with INPLASY (202170013). DOI number is 10.37766/inplasy2021.7.0013. Registration Date:2021.7.06.

## Background

The mental health of adolescents has become an increasingly serious public health problem worldwide [1]. Depression is a common mental illness in adolescents, with a prevalence of about 4.5% [2]. Depression seriously endangers adolescents’ physical and mental health, academic performance, and interpersonal relationships [3]. In severe cases, these adolescents may even commit suicide [4]. In recent years, the incidence of depression in China has continued to rise, and adolescents account for a prominent proportion of patients in the clinic [5, 6]. When adolescents with depressive symptoms or negative emotions do not receive timely intervention, they risk developing depression [7]. On August 31, 2020, China’s National Health Commission released the “Working Plan for Exploring Special Services for the Prevention and Treatment of Depression” [8]. The plan stated that high schools should add depression screening to student health examinations, as results show that many students have depressive symptoms. Effective strategies to reduce depressive symptoms in adolescents are needed.

The effect of exercise on depression has become a research hotspot in recent years [9, 10]. Cross-sectional studies over the past 30 years have suggested that low physical activity is an important risk factor for the development of depression [11, 12]. Prospective cohort studies have suggested that regular exercise reduces the risk of developing depression [13, 14]. Human and animal experiments showed that exercise can exert an antidepressant effect by increasing mitochondrial activity in brain neurons, stimulating the secretion of monoamine neurotransmitters, increasing the concentration of neurotrophic factors, inhibiting the overexpression of inflammatory factors, and regulating the expression of microRNAs [15]. It can also reduce depressive symptoms by improving self-efficacy, reducing negative emotions, and stimulating positive behaviors in depressed patients [16]. RCTs have also shown that structured exercise programs can effectively alleviate depressive symptoms, and there is a dose–response relationship [17, 18]. Studies have found that moderate- and high-intensity physical exercise can have a positive effect in the treatment of mild and moderate depression [19]. For adults, depression is usually regarded as the mental health problem most likely to be positively affected by exercise [20].

Antidepressant medication may be associated with side effects such as weight gain, sleep disturbance, and reproductive dysfunction which can be disturbing for adolescents [21]. Psychotherapy is more resource-intensive and is associated with perceived stigma from attending the therapist [22]. Comparatively, physical exercise is more cost-effective. It is convenient to implement within the community and can potentially have wider reach and participation [23]. There is less research on exercise interventions to treat depression in adolescents compared to adults, especially examining the moderating effects of exercise-related variables (e.g., exercise type, exercise program duration, exercise session duration, exercise intensity, and exercise frequency) [24]. Although RCTs in children and young people have shown that physical exercise can relieve depression and depressive symptoms [25, 26], the dose–response relationship remains unclear. Given this, this study aimed to systematically summarize the effect of physical exercise on adolescent depression and to clarify the dose–response relationship between physical exercise and depressive symptoms in adolescents.

## Methods

**Fig. 1:**
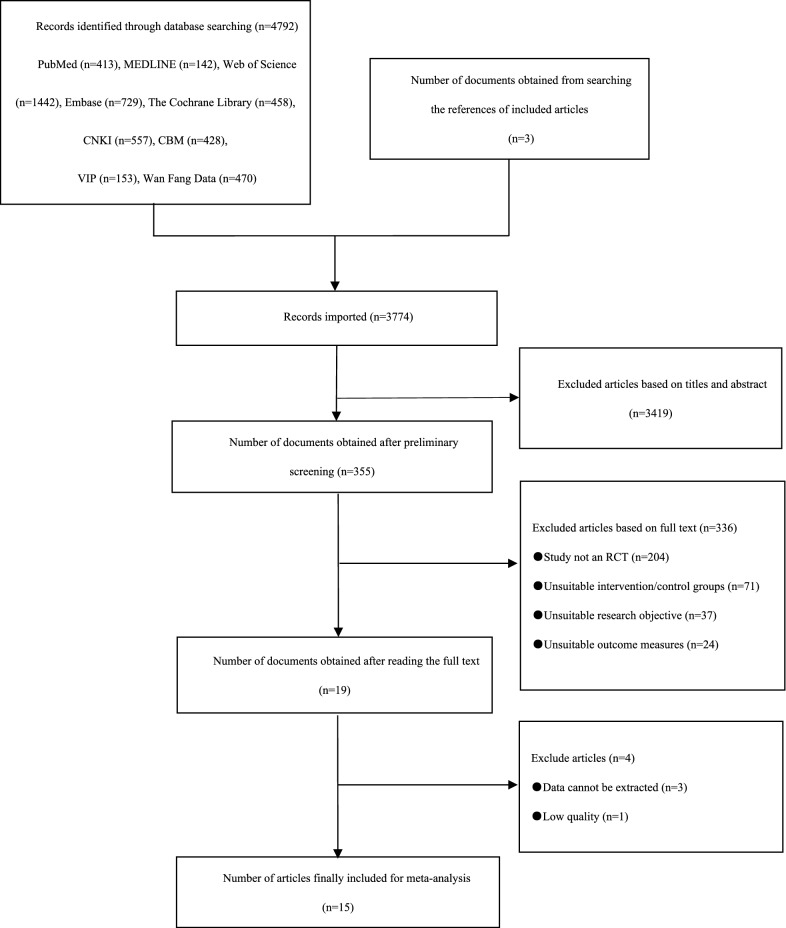
Article screening flow chart

The study adhered to the Preferred Reporting Items for Systematic Reviews and Meta-Analyses (PRISMA) 2020 guidelines (Fig. 1) [27].

### Inclusion criteria

Based on the PICOS (participants, interventions, comparisons, outcomes, study design) process used in evidence-based medicine [28], the inclusion criteria were as follows: (1) Participants: adolescents aged 12–18 diagnosed with depression (based on the Diagnostic and Statistical Manual of Mental Disorders [DSM-IV/5] or International Classification of Diseases [ICD-10]) or assessed to have depressive symptoms. (2) Intervention: the experiment group received a structured exercise program (aerobic exercise, resistance + aerobic exercise, or physical and mental exercise such as yoga [29]) compared with the control group [30]; if there were multiple experimental groups in the study, only the group with exercise intervention was included; if there had been multiple independent experiment groups in the same article, it was counted as multiple independent comparisons as well. (3) Comparison: The control group had no exercise intervention. (4) Outcomes: An internationally recognized depressive symptom-related scale was used, and its score was used as the study outcome. (5) Study design: randomized controlled trials (RCTs); original peer-reviewed Chinese or English papers.

### Exclusion criteria

The exclusion criteria were as follows: (1) Participants: Any identified physical or non-depressive mental illness (such as cancer, diabetes, overweight/obesity, or anxiety disorders). Studies which did not provide any information on the participants’ characteristics were excluded. (2) Interventions: Use of combined interventions, such as exercise combined with music therapy or cognitive training; lack of description of the physical exercise in the intervention design (type; program duration; session duration; intensity; or frequency). (3) Comparisons: Lack of a control group or control interventions that significantly increased cardiovascular activity. (4) Outcome Measures: Depressive symptom scores not evaluated pre- and post-intervention; Data could not be extracted or original data could not be obtained by contacting the corresponding author. (5) Study design: Scores on the modified PEDro scale of less than 4.

### Literature retrieval strategy

Nine databases were searched, comprising PubMed, Web of Science, The Cochrane Library, Embase, MEDLINE, China National Knowledge Infrastructure (CNKI), Chinese Biomedical Literature Database (CBM), VIP database, and Wanfang Database. The search was conducted from database inception to May 1, 2021.

The search strategy involved a combination of subject terms and free words, and was finalized after repeated checks. Chinese search terms included: adolescents, junior high school students, high school students; exercise, aerobic exercise, resistance exercise, high-intensity interval, physical and mental exercise, yoga, dance, aerobics, running, walking; depression, depression symptoms, depressive symptoms, negative emotions; randomized controlled trials. As an example of the English search terms, the PubMed search strategy was as follows:

#1 Adolescent [MeSH Terms] OR Adolescents [Title/Abstract] OR Adolescence [Title/Abstract] OR Teenager [Title/Abstract] OR Teenagers [Title/Abstract] OR Teen [Title/Abstract] OR Teens [Title/Abstract] OR Youth [Title/Abstract] OR Youths [Title/Abstract];

#2 Exercise [MeSH Terms] OR Exercise [Title/Abstract] OR Aerobic Exercise [Title/Abstract] OR Resistance Exercise [Title/Abstract] OR High-Intensity Interval Training [Title/Abstract] OR Mind–Body Exercise [Title/Abstract];

#3 Depression [MeSH Terms] OR Depressive Disorder [Title/Abstract] OR Depressive Symptom [Title/Abstract] OR Emotional Depression [Title/Abstract] OR Negative Emotion [Title/Abstract];

#4 randomized controlled trial [MeSH Terms].

#5 #1 and #2 and #3 and #4.

### Literature screening, data extraction, and quality evaluation

#### Literature screening

EndNote X9 software was used to remove the duplicates from the search results. Thereafter, two authors (both of whom were experienced researchers in the field) independently screened the literature according to the inclusion and exclusion criteria. First, the titles and abstracts were read to preliminarily screen the articles, and the articles that did not meet the inclusion and exclusion criteria were deleted and recorded. Second, the full text of the remaining articles was downloaded, read, and reviewed to re-screen the articles. If there was a disagreement between both authors, a third author would review and decide whether to include the study.

#### Data extraction

Two authors used a pre-designed data extraction form to extract the following data and record it: (1) Basic article information: first author’s name, study country, and year of publication. (2) Basic information: depression type (depression or depressive symptoms), age, sex ratio, and sample size. (3) Physical exercise variable (e.g., exercise type, exercise program duration, exercise session duration, exercise intensity, and exercise frequency).

#### Quality evaluation

Two authors used a modified version of the PEDro scale [31] to evaluate the methodological quality of the included studies. If there was a disagreement, a third author evaluated the issue, which was discussed until a consensus was reached. The 10 items on the scale include "eligibility criteria", "random allocation", "allocation concealment", "baseline similarity between groups", "exercise intensity control", "blinded outcome evaluation", and "dropout rate < 15%", "intention-to-treat analysis", "Statistical analysis comparing groups", "point and variability measures". If the relevant standard was clearly met, the item was scored as 1 point; if the relevant standard was not clearly met or not mentioned, the item was scored as 0. The highest score that could be achieved was 10 points, so < 4, 4–5, 6–8, and 9–10 indicated low, medium, good, and high quality, respectively.

### Statistical analysis

The statistical analysis was conducted in Stata 16.0 software. The outcome variables were continuous, and mean ± standard deviations (SD) were extracted for each included comparison. There were no significant differences in the outcome variables between the groups in each comparison at baseline. At the end of the experiment, we chose scale scores of both the intervention group and the control group as the effect size, which reflects the intervention effect. Due to the use of multiple depression scales among the included articles, standardized mean difference (SMD) was used as the effect size for analysis, with 0.2, 0.5, and 0.8 indicating small, moderate, and large effect sizes, respectively [32]. Heterogeneity was quantified by I^2^ (with 75%, 50%, and 25% indicating high, medium, and low degrees of inter-study heterogeneity, respectively [33]) and Cochran’s Q test p value. If there is publication bias among the included articles, the trim and fill method was used to correct for asymmetry.

## Results

### Literature retrieval results

As shown in Fig. 1, a total of 4792 articles were obtained by searching PubMed (n = 413), MEDLINE (n = 142), Web of Science (n = 1442), Embase (n = 729), The Cochrane Library (n = 458), CNKI (n = 557), CBM (n = 428), VIP database (n = 153), and Wanfang Database (n = 470). Additionally, 3 articles were obtained by searching the references of the included articles. After deduplication, 3774 articles were obtained. After preliminary screening, 355 articles were obtained. After re-screening the articles by reading the full texts and excluding articles due to unsuitable study design (not an RCT) intervention/control groups, research objective, or outcome measures, 19 articles were obtained. After excluding articles with unavailable data or low-quality articles, 15 articles were included in the meta-analysis.

### Characteristics of included literature

As shown in Table 1, 15 articles were included, with 19 comparisons. The publication year was 1982 to 2017. There were 1331 participants, ranging from 24 to 209 per article. The mean age of the participants was 15.90 ± 1.23 years old. The comparisons were conducted in 8 countries: the United States (n = 8) [34–40], Iran (n = 3) [41, 42], Germany (n = 2) [43], Australia (n = 2) [44], the United Kingdom (n = 1) [45], South Korea (n = 1) [46], Chile (n = 1) [47], and Colombia (n = 1) [48]. Regarding depression type, 6 comparisons were on depression and 13 were on depressive symptoms. Regarding recruitment, 9 comparisons involved participants recruited from the following special organizations: inpatient department (n = 3), mental health center (n = 2), community outpatient clinic (n = 1), juvenile detention center (n = 2), and school for young offenders (n = 1). Additionally, 10 comparisons involved exercise interventions carried out in school (5 in junior high school and 5 in high school).

**Table 1:** Characteristics of included literature

Name	Country	Population	Mean age (year)	Sample size N (female %)	Exercise group				Control group	Outcome	Effect of exercise and follow-up points
					Type	Exercise program duration (week)	Exercise session duration (min)	Frequency (sessions/week)			
Brown et al. (1992)	United States	1. Diagnosed at mental health center; 2. Depression	15.60	27 (41%)	Aerobic exercise (jogging)	9	45	3	Conventional treatment	BDI	Significant ▲: 11; △: 5 No adverse events
Roshan et al. (2011)	Iran	1. Diagnosed at hospital; 2. Depression	16.87	24 (100%)	Aerobic exercise^①^ (walking in water)	6	30	3	Conventional treatment	HAMD	Significant ▲: 0; △: 0 No adverse events
Hughes et al. (2013)	United States	1. Diagnosed at outpatient clinic; 2. Depression	17.00	26 (42%)	Aerobic exercise (jogging/power bike)	12	30	3	Conventional treatment	CDRS-R	1. Not significant at 3/12 weeks and significant at 6/9 weeks 2. Follow-up: 24/48 weeks 3. ▲: 0; △: 0 4. No adverse events
Carter et al. (2015)	United Kingdom	1. Diagnosed at mental health center; 2. Depression	15.40	87 (78%)	Aerobic exercise^②^ (aerobics)	6	45	3	Conventional treatment	CDI-2	1. Not significant 2. Follow-up: 24 weeks (significant) 3. ▲: 8; △: 14 4. No adverse events
Wunram et al. (2017a)	Germany	1. Diagnosed at inpatient department; 2. Depression	15.80	44 (77%)	Whole-body muscle vibration training	6	30	4	Conventional treatment	DIKJ	1. Not significant 2. Follow-up: 14 /26 weeks (the second of which was significant) 3. ▲: 3; △: 6 4. No adverse events
Wunram et al. (2017b)	Germany	1. Diagnosed at inpatient department; 2. Depression	15.90	43 (72%)	Aerobic exercise (power bike)	6	30	4	Conventional treatment	DIKJ	1. Not significant 2. Follow-up: 14 /26 weeks (the second of which was significant) 3. ▲: 3; △: 6 4. No adverse events
Hilyer et al. (1982)	United States	1. Recruited at school for young offenders; 2.Depressive symptoms	17.00	60 (-)	Resistance + aerobic exercise	20	90	3	Conventional treatment	BDI	Significant ▲: 0; △: 0 No adverse events
MacMahon et al. (1988a)	United States	1. Recruited at juvenile detention center; 2.Depressive symptoms	15.60	39 (-)	Aerobic exercise^③^ (aerobics)	12	40	3	Regular activities	BDI	Significant ▲: 0; △: 0 No adverse events
MacMahon et al. (1988b)	United States	1. Recruited at juvenile detention center; 2.Depressive symptoms	17.20	30 (-)	Aerobic exercise^③^ (aerobics)	12	40	3	Regular activities	BDI	Significant ▲: 0; △: 0 No adverse events
Bonhauser et al. (2005)	Chile	1. 9th grade public junior high school students; 2. Depressive symptoms	15.53	198 (52%)	Aerobic exercise	40	90	3	Regular activities	HADS	Not significant ▲: 8; △: 7 No adverse events
Mohammadi. (2011a)	Iran	1. Public high school students; 2. Depressive symptoms	16.60	60 (-)	Aerobic exercise (group aerobic exercise)	8	75	3	Conventional treatment	BDI	Significant ▲: 0; △: 0 No adverse events
Mohammadi. (2011b)	Iran	1. Public high school students; 2. Depressive symptoms	16.60	60 (-)	Aerobic exercise (personal aerobic exercise)	8	75	3	Conventional treatment	BDI	Significant ▲: 0; △: 0 No adverse events
Jeong et al. (2005)	Korea	1. Girls’ high school students; 2. Depressive symptoms	16.00	40 (100%)	Aerobic exercise (aerobic dance)	12	45	3	Conventional treatment	SCL-90-R	Significant ▲: 0; △: 0 No adverse events
Khalsa et al. (2012)	United States	1. 11/12th grade senior high school students in rural school; 2. Depressive symptoms	16.80	121 (42%)	Self-designed yoga	11	30	2	Regular activities	POMS-SF	Not significant ▲: 3; △: 17 No adverse events
Noggle et al. (2012)	United States	1. 11/12th grade senior high school students in rural school; 2. Depressive symptoms	17.20	51 (57%)	Kripalu Yoga	10	40	3	Regular activities	POMS-SF	Not significant ▲: 0; △: 0 No adverse events
Velasquez et al. (2015)	Colombia	1. Public junior high school students; 2. Depressive symptoms	13.20	125 (-)	Satyananda Yoga	12	120	2	Regular activities	SDQ	Not significant ▲: 11; △: 0 No adverse events
Butzer et al. (2017)	United States	1. 7th grade public junior high school students; 2. Depressive symptoms	12.64	209 (63%)	Kripalu Yoga	16	45	2	Regular activities	BRUMS	1. Significant 2. Follow-up: 24/48 weeks 3. ▲: 4; △: 0; 4. No adverse events
Costigan et al. (2016a)	Australia	1. Public junior high school students; 2. Depressive symptoms	15.65	43 (-)	Aerobic exercise^④^ (aerobic running and jumping)	8	8	3	Regular activities	K-10	Not significant ▲: 2; △: 0; No adverse events
Costigan et al. (2016b)	Australia	1. Public junior high school students; 2. Depressive symptoms	15.55	44 (-)	Resistance + aerobic exercise^④^	8	8	3	Regular activities	K-10	Not significant ▲: 1; △: 0; No adverse events

*BDI * Beck Depression Inventory, *HAMD* Hamilton Depression Scale, *CDRS- R* Childhood Depression Rating Scale-Revised; *CDI-2* Children’s Depression Inventory-2, *DIKJ* Depressionsinventar für Kinder und Jugendliche (German version of CDI-2), *HADS* Hospital Anxiety and Depression Scale, *SCL-90-R* Symptom Checklist-90 -Revised; *POMS-SF* Profile of Mood States-Short Form, *BRUMS* Brunel University Mood Scale, *SDQ* Strengths and Difficulties Questionnaire, *K10* Kessler Psychological Distress Scale. Number of dropouts in experiment group: ▲; Number of dropouts in control group: △. Exercise intensity: ① 60–70% maximum heart rate (HRmax); ② Self-selected intensity, ≤ 80% HRmax; ③120–160 beats/min; ④ ≥ 85% HRmax

“a” represents the first comparison. b represents the second comparison.

The exercise programs mainly involved aerobic exercise, resistance + aerobic exercise, or yoga (though one article involved whole-body muscle vibration). The total exercise program duration for adolescents with depression ranged from 6 to 12 weeks, the exercise session duration ranged from 30 to 45 min, exercise intensity was moderate intensity (1 comparison) and optional intensity (1 comparison), and the exercise frequency ranged from 3 to 4 times per week. The total exercise program duration for adolescents with depressive symptoms ranged from 6 to 40 weeks, the exercise session duration ranged from 8 to 120 min, exercise intensity was moderate intensity (2 comparisons) and self-selected intensity (2 comparisons), and the frequency ranged from 2 to 3 times per week.

Nine comparisons (depression: 3; depressive symptoms: 6) showed that there was no significant difference in depression scores between the exercise and control groups at the end of the study, while 10 comparisons (depression: 3; depressive symptoms: 7) showed significant differences. Five comparisons (depression: 4; depressive symptoms: 1) included a follow-up period after the experiment. Two of these comparisons (depression: 1; depressive symptoms: 1) showed that the depression scores during the follow-up period were not significantly different between the exercise and control groups, while 3 comparisons (depression: 3) showed significant differences. There were no adverse events among the included studies. The mean dropout rate of the exercise group was 8.33% and that of the control group was 9.21%, with no significant difference (t = -0.18, p = 0.86).

### Methodological quality evaluation

As shown in Table 2, the PEDro score among the 15 included articles was 5–8 points. There were 4 medium and 11 high-quality articles, with a mean of 6 points. The overall research quality was good. All articles mentioned “eligibility criteria”, “random allocation”, “baseline similarity between groups”, “statistical analysis comparing groups”, and “point and variability measures”. Additionally, 4 articles mentioned “exercise intensity control”, 1 article mentioned “blinded outcome evaluation”, 2 articles mentioned using “intention-to-treat analysis”, and 7 articles mentioned “dropout rate < 15%” (Table 2).

**Table 2:** Methodological quality of included articles

Article	Eligibility criteria	Random allocation	Allocation concealment	Baseline similarity between groups	Exercise intensity control	Blinded outcome evaluation	Dropout rate < 15%	Intention-to-treat analysis	Statistical analysis comparing groups	Point and variability measures	Total score
Brown et al. (1992)	1	1	0	1	0	0	0	0	1	1	5
Roshan et al. (2011)	1	1	0	1	1	0	1	0	1	1	7
Hughes et al. (2013)	1	1	0	1	0	0	1	0	1	1	6
Carter et al. (2015)	1	1	1	1	1	1	0	0	1	1	8
Wunram et al. (2017)	1	1	0	1	0	0	0	1	1	1	6
Hilyer et al. (1982)	1	1	0	1	0	0	0	0	1	1	5
MacMahon et al. (1988)	1	1	0	1	1	0	0	0	1	1	6
Bonhauser et al. (2005)	1	1	0	1	0	0	0	0	1	1	5
Mohammadi (2011)	1	1	0	1	0	0	1	0	1	1	6
Jeong et al. (2005)	1	1	1	1	0	0	0	0	1	1	6
Khalsa et al. (2012)	1	1	0	1	0	0	1	1	1	1	7
Noggle et al. (2012)	1	1	0	1	0	0	0	0	1	1	5
Velasquez et al. (2015)	1	1	0	1	0	0	1	0	1	1	6
Butzer et al. (2016)	1	1	0	1	0	0	1	0	1	1	6
Costigan et al. (2016)	1	1	0	1	1	0	1	0	1	1	7

### Meta-analysis of the impact of exercise on depression in adolescents

#### Meta-analysis results

**Fig. 2:**
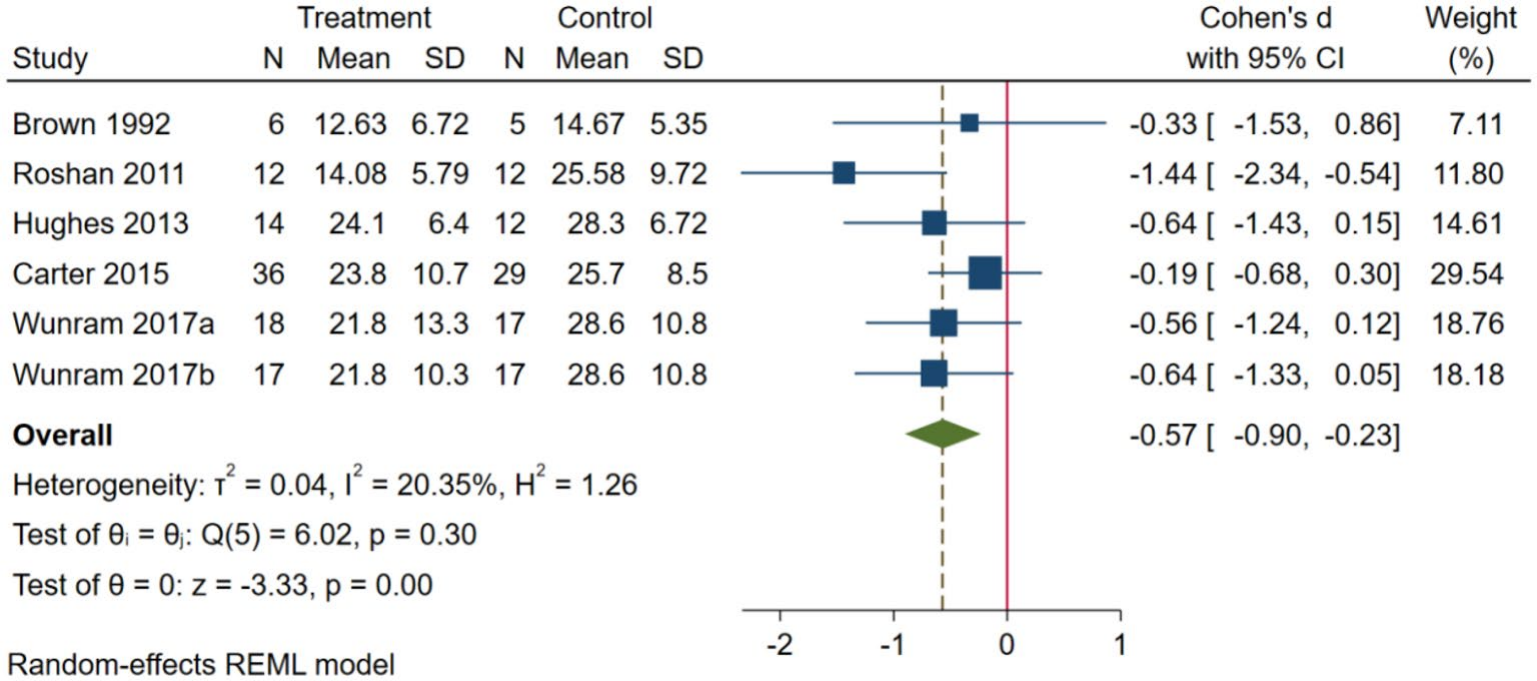
Forest plot of the effect of exercise on adolescents with depression

**Fig. 3:**
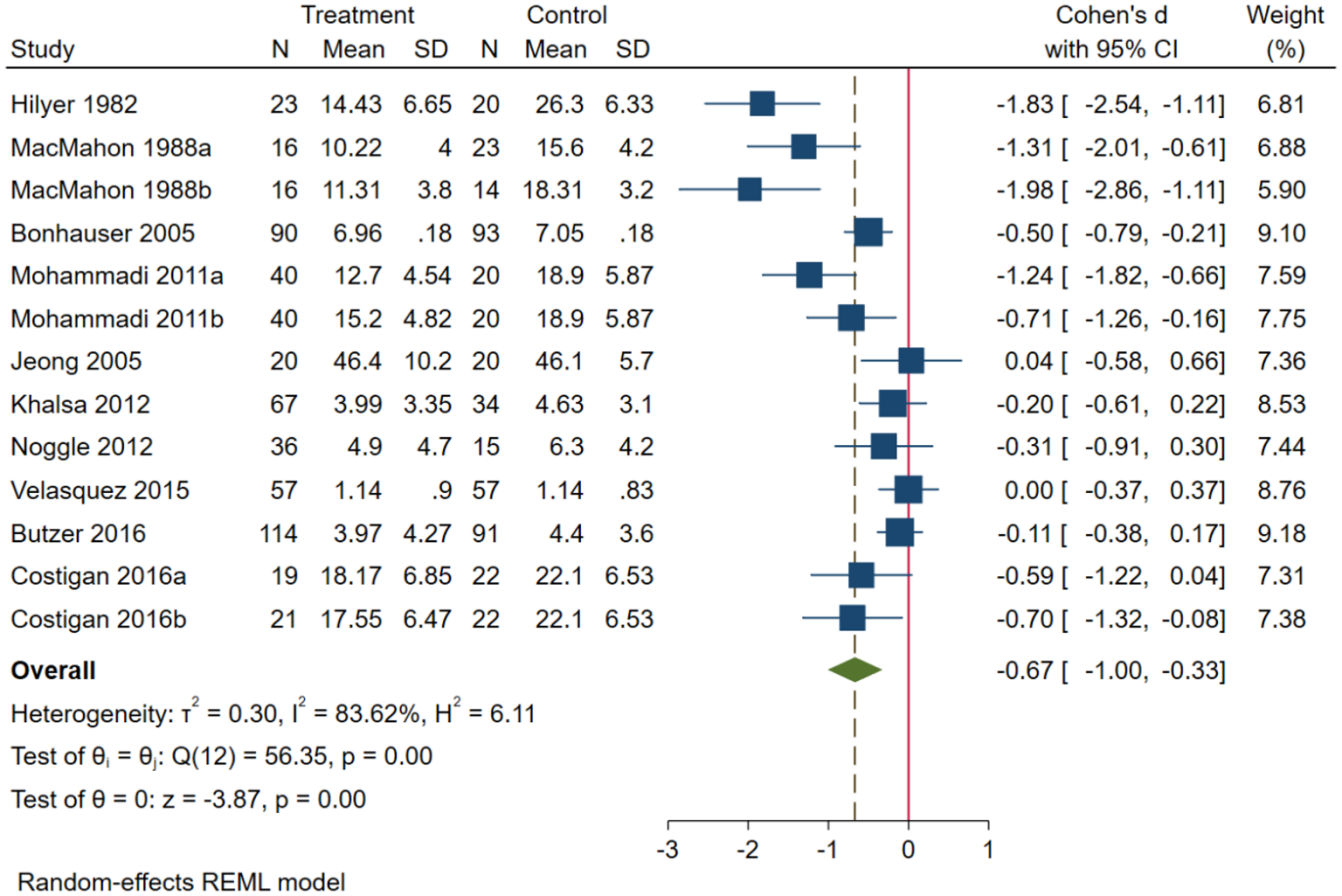
Forest plot of the effect of exercise on adolescents with depressive symptoms

A total of 19 studies included I^2^ = 75.05%, combined effect size SMD = − 0.64, 95% CI (− 0.89, − 0.39, P < 0.01). The results showed that post-intervention, subjects in the intervention group showed more significant reduction in depressive symptoms than the control group, with a moderate effect size. As shown in Fig. 2, adolescents with depression I^2^ = 20.35%, combined effect size SMD = -0.57, 95% CI (− 0.90, − 0.23, P < 0.01), there is low heterogeneity. As shown in Fig. 3, adolescents with depressive symptoms I^2^ = 83.62%, combined effect size SMD = − 0.67, 95% CI (− 0.90, − 0.23), P < 0.01, there is a high degree of heterogeneity.

### Sensitivity analysis

**Fig. 4:**
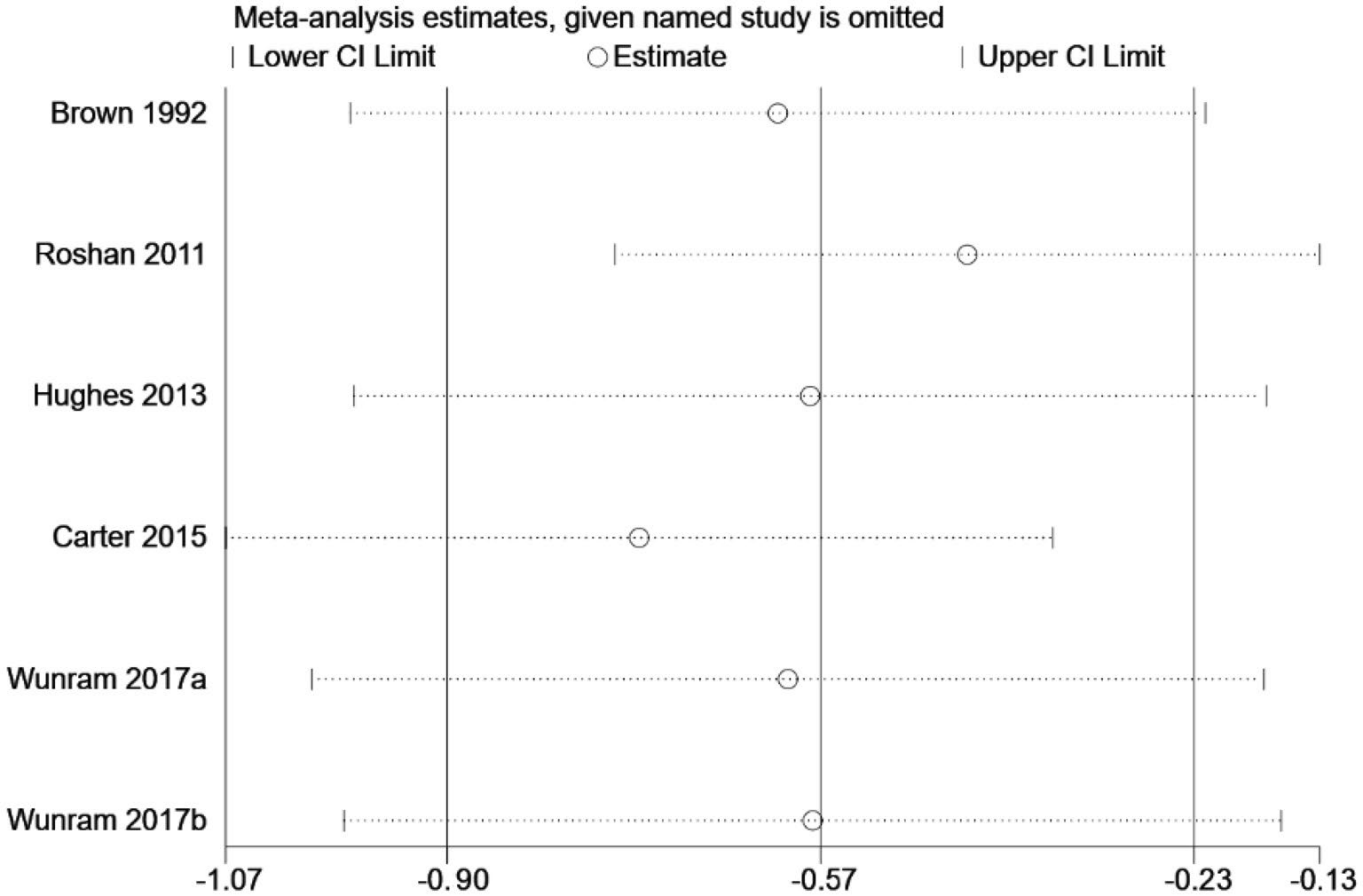
Sensitivity analysis of the effect of exercise on depression in adolescents

**Fig. 5:**
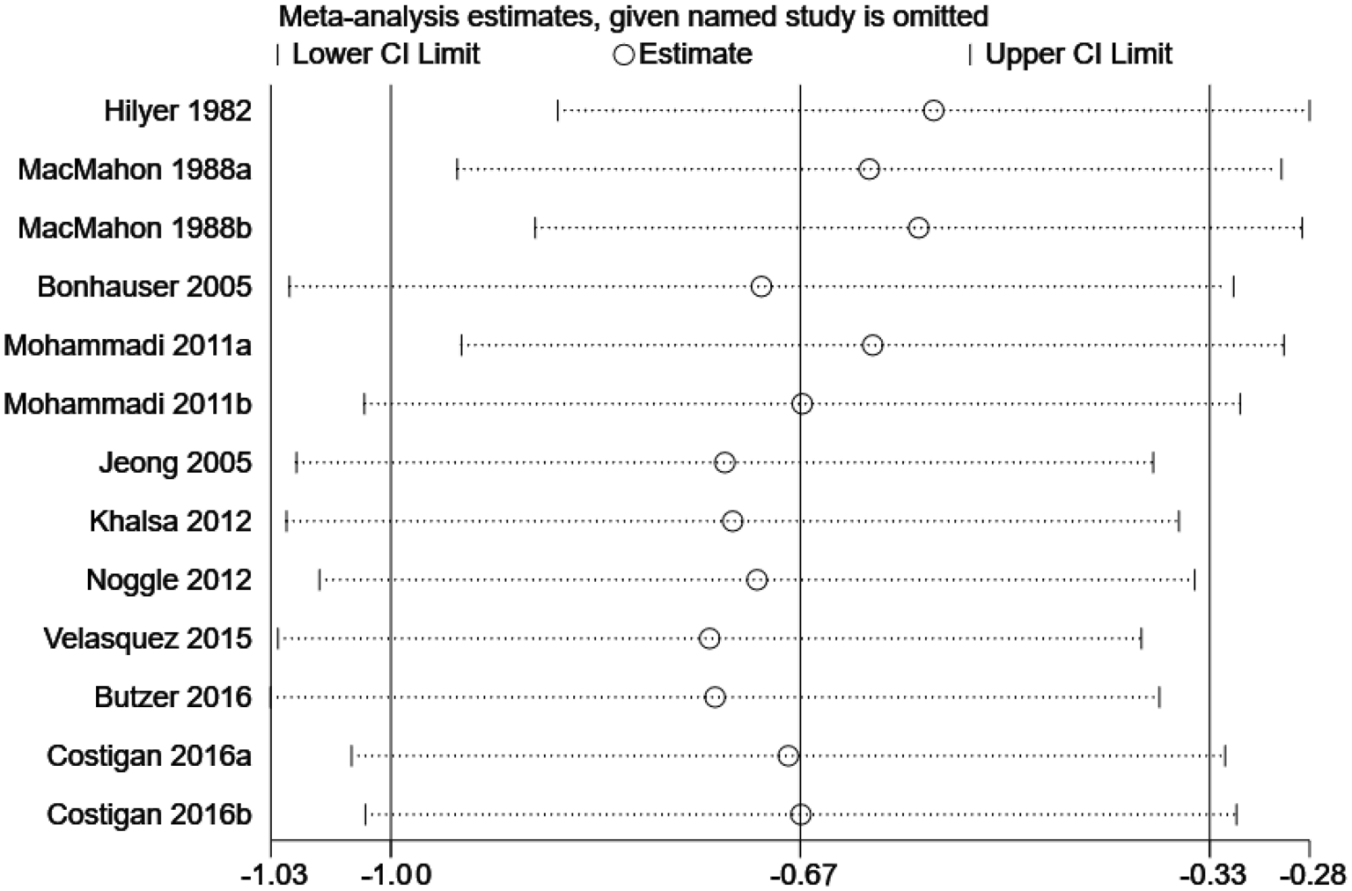
Sensitivity analysis of the effect of exercise on depressive symptoms in adolescents

In order to explore whether the heterogeneity between studies is caused by a single study, Stata 16.0 software was used for sensitivity analysis [49]. As shown in Figs. 4 and 5, the effect size of the depression group and the depressive symptom group were not significantly changed after eliminating single studies one by one, indicating that the study results were relatively stable.

### Subgroup analysis

As shown in Table 3, to further explore the source of heterogeneity, a subgroup analysis of potential moderating variables was performed. Regarding exercise type, the effect of aerobic exercise among adolescents with depression was significant. The effects of aerobic exercise and resistance + aerobic exercise among adolescents with depressive symptoms were also significant, while the effect of yoga was not significant. There was a high degree of heterogeneity in the aerobic exercise and resistance + aerobic exercise subgroups among adolescents with depressive symptoms. Regarding program duration, the effect of continuous intervention for 6 weeks in adolescents with depression was significant, while the effect of continuous intervention for 9–12 weeks was not significant. For adolescents with depression symptoms, the effect of continuous intervention for 8 weeks was significant, while the effects of continuous intervention for 10–12 and > 12 weeks were not significant. There was a high degree of heterogeneity in the > 8-week subgroup among adolescents with depressive symptoms. Regarding exercise session duration, the effect of 30 min of exercise in adolescents with depression was significant, while 45 min was not significant. For adolescents with depressive symptoms, the effect of 75–120 min was significant, but the effect of 30–45 min was not significant. There was a high degree of heterogeneity in the 30–45 and 75–120 min subgroups among adolescents with depressive symptoms. Regarding exercise frequency, 3–4 times/week among adolescents with depression was significant, and 4 times/week was better. The effect of 3 times/week in adolescents with depressive symptoms was significant, while 2 times/week was not. The effect of exercise 3 times/week among adolescents with depressive symptoms was highly heterogeneous. Moderate and high intensity exercise had a significant effect on depressive symptoms among adolescents with moderate intensity interventions producing the greater benefit.

**Table 3:** Subgroup analyses of the effect of exercise on depression in adolescents

Variable	Categories		Number of comparisons (number of participants)	Heterogeneity test results		Meta-analysis results	
				Q value	I^2^ value	SMD effect size (95% CI)	P value
Exercise type	Depression	Aerobic	5 (160)	6.02	35.29%	− 0.59 (− 1.01, -0.17)	0.01
		Whole-body muscle vibration training	1 (35)	–	–	–	–
	Depressive symptoms	Aerobic	7 (453)	21.70	76.91%	− 0.85 (− 1.29, -0.40)	< 0.01
		Resistance + aerobic exercise	2 (86)	4.78	79.07%	− 1.25 (− 2.40, -0.10)	0.03
		Yoga	4 (471)	0.91	0%	− 0.12 (− 0.30, 0.07)	0.22
Exercise program duration (weeks)	Depression	6	4 (158)	5.84	47.46%	− 0.61 (− 1.07, 0.15)	0.01
		9–12	2 (37)	0.18	0%	− 0.55 (− 1.21, 0.11)	0.10
	Depressive symptoms	8	4 (204)	2.79	0%	− 0.82 (− 1.11, -0.52)	< 0.01
		10–12	6 (375)	25.97	86.46%	− 0.57 (− 1.18, 0.04)	0.07
		> 12	3 (431)	20.12	95.18%	− 0.76 (− 1.73, 0.21)	0.12
Exercise session duration (min)	Depression	30	4 (119)	2.72	0%	− 0.76 (− 1.13, -0.38)	< 0.01
		45	2 (76)	0.04	0%	− 0.21 (− 0.67, 0.24)	0.35
	Depressive symptoms	30–45	6 (466)	25.32	87.31%	− 0.58 (-1.18,0.01)	0.06
		75–120	5 (460)	26.51	87.84%	− 0.80 (− 1.38, -0.21)	0.01
Exercise frequency (sessions/week)	Depression	3	4 (126)	5.80	48.59%	− 0.58 (-1.11, -0.05) )	0.03
		4	2 (69)	0.03	0%	− 0.60 (-1.08, -0.12)	0.01
	Depressive symptoms	2	3 (420)	0.49	0%	− 0.10 (-0.29, 0.10)	0.33
		3	10 (590)	3.74	76.47%	− 0.87 (-1.25, -0.49)	< 0.01
Exercise intensity	Depression	Moderate	1 (24)	–	–	–	–
		Self-selected	1 (65)	–	–	–	–
	Depressive symptoms	Moderate	2 (69)	1.39	28.18%	− 1.59 (-2.24, -0.94)	< 0.01
		High	2 (84)	0.06	0	− 0.65 (-1.08, -0.21)	< 0.01

### Publication bias

The Egger regression method was used to assess publication bias regarding the included articles [50]. Egger’s test results showed that there was no publication bias in the depression group (t = − 1.42, P = 0.23, 95% CI − 6.45, 2.09). In the depressive symptom group, the result showed likelihood of publication bias (t = − 3.12, P = 0.01, 95% CI − 6.87, − 1.19). The reason for publication bias is that more positive results than negative results were included [51]. The trim and fill method [52] was used to identify and correct the asymmetry caused by publication bias. The results show that no sample needed to be corrected or recalculated among the experimental samples. The random-effects model calculates the point estimate of the combined RR and 95% CI was − 0.65 (− 0.87, -0.58) after trim-and-fill, and the effect size RR difference before and after trim-and-fill did not change significantly, suggesting that publication bias has little effect on the results, and the meta-analysis results are relatively robust.

## Discussion

This study showed that exercise has a moderate effect on alleviating depressive symptoms in adolescents, which is consistent with the results from a previous meta-analysis [53]. A previous meta-analysis on the use of exercise to treat depression in children and adolescents also showed that exercise had a small-to-moderate effect [21], but due to the heterogeneity among the patients, the authors stated that there was insufficient evidence to prove the benefits of exercise. Additionally, two meta-analyses of young people (4–25 years old) found that exercise has a moderate-to-large effect [54, 55]. However, in addition to RCTs, these two meta-analyses included quasi-experimental and observational studies, and the overall methodological quality was low. In addition, previous meta-analyses covered participants with a wide range of ages [21, 50, 51]. Given the biological and psychological differences between children and adolescents [56], this may have had a confusing effect on the summary results. Therefore, unlike these previous meta-analyses, our meta-analysis only included adolescents aged 12–18 years who had been diagnosed with depression or had been assessed to have significant depressive symptoms. We also excluded studies which recruited individuals with comorbid diseases closely related to depression. Furthermore, we explored the moderating effects of exercise-related variables in order to better assess the dose–response relationship of exercise. The sensitivity analysis and publication bias test suggested that the results were highly stable.

In this meta-analysis, 5 comparisons involved follow-up results, 3 of which suggested that exercise had a sustained benefit (at about 6 months) after the intervention. First, Carter et al. found that the depressive symptoms at week 24 in the 6-week aerobics exercise group were significantly lower than those in the control group [41]. Second, the two comparisons by Wunram et al. (one involving aerobic exercise and the other involving whole-body muscle vibration) both found that there was a significant difference at week 26 in depression scores between the exercise and control groups. The depression remission rate in both the exercise groups (67.8%) was significantly higher than that in the control group (26.8%) [39]. The authors explained that this may be related to the participants maintaining regular exercise after the intervention [57]. Due to the limited number of follow-up studies, short timeframes, and lack of continuous measurement, larger follow-up studies are still needed to further explore the sustainability of the effectiveness of exercise interventions to reduce depression in adolescents.

In addition, research has shown that 80% of depressed adolescents refuse to be treated again due to side effects or stigma after the first psychological or drug treatment [58]. In contrast, none of our included articles reported any adverse events during the interventions. A meta-analysis of the effectiveness of treatment for depression in adolescents found that the dropout rates regarding psychological and drug therapy among adolescents were approximately 23% and 45%, respectively [59]. In contrast, in our meta-analysis, the mean dropout rates were 8.33% and 9.21% in the exercise and control groups, respectively, with no significant difference (t = − 0.18, p = 0.86). Given that exercise has similar benefits to psychological and drug therapy, the high compliance among adolescents with exercise, and the many benefits of exercise in the growth and development stages [60], the use of exercise to prevent and treat depression in adolescents is feasible and acceptable.

At present, most studies report that the cause of depression is related to the dysfunction of neurotransmitters such as serotonin (5-hydroxytryptamine), dopamine, and norepinephrine [61]. In rats, swimming exercises over 10 weeks significantly increased the levels of serotonin, dopamine, and norepinephrine in the hippocampus [62]. Voluntary running exercises for 8 weeks significantly increased the levels of dopamine and its metabolites in the prefrontal cortex and striatum of rats [63]. After a 6-week jogging intervention for patients with depression, the plasma levels of gamma-aminobutyric acid (GABA) increased and the depression symptoms decreased [64]. Six months of moderate-intensity aerobic exercise significantly increased the prefrontal cortex gray matter volume in patients with depression (decreased gray matter volume is a key physiological sign of depression [65]), which was directly proportional to the amount of exercise [66]. In addition, exercise among patients with depression upregulated brain-derived neurotrophic factor (BDNF), which stimulates and mediates neurogenesis and regulates depressive behavior [67], in the hippocampus and cortex, promoted hippocampal neurogenesis, and significantly enhanced synaptic plasticity [68]. In addition, exercise alleviated hypothalamic–pituitary–adrenal feedback regulation obstacles by modulating cortisol and IL-6 levels, and thereby improved depression [69].

Current research generally indicates that for people with depressive symptoms, physical exercise is as effective as antidepressant drugs and psychotherapy and, for patients with depression, exercise can be used as a supplement to traditional therapy [70–72]. Walking every day for 10 days, with an 80% maximum heart rate (HRmax), significantly reduced the Bech–Rafaelsen Mania Scale (BRMS) score of patients with major depression [73]. Cycling at 70–80% HRmax for 12 weeks reduced the symptoms of individuals with depressive symptoms, and improved maximum oxygen uptake and visuospatial memory [74]. Aerobic exercises in physical education classes significantly reduced adolescents’ impulsivity, anxiety, drug abuse [36, 38]. Studies have found that the effect of physical exercise on depression is influenced by the severity of depression, and is significantly negatively correlated with the level of depression [75, 76]. This is because patients with depression usually have a low mood for more than 2 weeks, which is accompanied by a decrease in hippocampus volume, structural changes in the prefrontal cortex, cingulate gyrus, and temporal lobe, as well as cognitive dysfunction [77]. The symptoms of general depression are usually mild, often manifested as lack of happiness, low self-esteem, pessimism, loneliness, and other negative emotions [78]. A single exercise session among individuals with depressive symptoms can significantly improve self-efficacy, thereby reducing negative emotions [79], while regular exercise for > 3 months can effectively reshape the central nervous system organization of patients with depression [80]. Participants' physical health may also be an important factor affecting the effectiveness of the intervention. Adolescents with mental or physical diseases such as obesity [81], chronic fatigue syndrome [82], attention deficit hyperactivity disorder [83], may differ from ordinary adolescents in their exercise tolerance. They are more likely to adhere to moderate- than to high-intensity exercise [84].

Exercise type may be the moderating factor that affects the effect of the intervention. At present, aerobic exercise is the most important type of exercise to treat depression [85]. Aerobic exercise increases the levels of vascular endothelial growth factor (VEGF) and insulin-like growth factor 1 (IGF-1) in the mouse brain, and increases the volume of the subventricular and subgranular zones in the hippocampal dentate gyrus, promoting the differentiation of hippocampal neurons [86]. In addition, aerobic exercise activates central nervous system neuroactive substances and BDNF in the brain [87]. Jeong et al. found that 12 weeks of aerobic dance in adolescents increased the plasma concentration of serotonin and decreased the concentration of dopamine, suggesting that the stability of the sympathetic nervous system increased [42]. Roshan et al. found that 6 weeks of aerobic walking in water significantly increased the 3-methoxy-4-hydroxyphenylglycol (MHPG) sulfate value in the urine of adolescents, and it was significantly negatively correlated with the HAMD score, suggesting that aerobic exercise reduced depressive symptoms in adolescents [37]. In addition, compared with aerobic exercise alone, resistance + aerobic exercise can produce complementary neurobiological and other physiological effects [88]. Regarding the two resistance + aerobic exercises included in our meta-analysis, Hilyer et al. found that the BDI score was significantly lower after a 20-week resistance + aerobic exercise program than that of the control group [32]. Second, Costigan et al. found that resistance + aerobic exercise slightly improved subjective well-being among adolescents, but not depressive symptoms [40]. As our meta-analysis only included 2 articles on resistance + aerobic exercise, its effect on depression in adolescents needs to be verified by multiple additional RCTs. In addition, due to the inherent listlessness of patients with major depression, it is sometimes difficult to motivate them to take active exercise[89]. Whole-body muscle vibration training was chosen as an auxiliary or supplementary exercise method. This kind of exercise can be performed on a high level of physical activity even with a low motivation to exercise[90]. Hyperactive HPA axis in patients with major depression is one of the important reasons for its onset[91]. There is some evidence that whole-body muscle vibration training can have a positive effect on maintaining stable cortisol secretion in adolescents with severe depression and reducing the activity of the HPA [43]. Due to the limited number of studies in this literature, vibration training currently needs stronger empirical evidence to investigate the effects of this approach in alleviating depression in adolescents.

In addition, our meta-analysis found that yoga did not significantly impact depression in adolescents. Yoga, as a physical and mental exercise based on body posture exercises, has been found to reduce anxiety and stress [92]. Yoga can improve negative emotions by regulating the hypothalamus–pituitary–adrenal axis and sympathetic nervous system, increasing thalamic GABA levels and reducing cortisol levels [93]. Of the 4 included yoga articles (satyananda yoga:1; kripalu yoga:2; self-designed yoga:1), only 1 study has a significant difference compared to the control group. Although these studies mentioned the type of yoga selected, they did not describe the specific intervention details during the implementation process. We can only roughly know that yoga elements include posture, breathing, relaxation and meditation. Among them, Kripalu Yoga is often described as "dynamic meditation"[94]. During practice, students need to pay more attention to the individual psychological feelings brought by yoga postures[95]. Therefore, students are required to maintain a gentle and introspective attitude throughout the practice. Each pose of Kripalu Yoga needs to be maintained for a long time in order to fully release the pent-up emotions[96]. Kripalu Yoga has achieved significant intervention effects in one study, which may be related to its emphasis on obeying the wisdom of the body[40]. The reason for the insignificant effect of yoga intervention may lie in the characteristics of yoga exercise, the duration of yoga experiment and experiment control. Adolescent males often resist participating in low-intensity exercise such as yoga and tend to choose more intense exercise types, so gender factors may impact the effect of yoga interventions [97]. For male students who do not like yoga, they would choose to use the word "active" to describe the purpose they want to pursue in their physical exercise. In yoga exercises, they feel more restrained [98]. As a complementary therapy for physical and psychological disorders, yoga has been extensively studied in adults [99]. Long-term follow-up showed that yoga led to delayed transformation, leading to improvement in long-term self-control in emotion, though the short-term effect was not significant [36]. In addition, the current practice of yoga among young people may limit its effectiveness due to the lack of specific standards for quality control of yoga implementation. Longer post-intervention follow-up studies on yoga interventions should be conducted, and the implementation processes should be clearly reported, so as to provide the best advice for young people on using yoga to relieve depression.

Exercise program duration, session duration, frequency, and intensity may moderate the effects of exercise. One study showed that there is an inverted U-shaped relationship between the exercise program duration and mental health symptom relief in adolescents [100]. Another study showed that maintaining regular exercise for 6–8 weeks significantly reduced negative emotions in adolescents, but with the prolongation of the program, the benefits did not significantly improve [101]. International public health physical activity guidelines stipulate that at least 150 min of moderate-intensity exercise should be performed every week to maintain health [102]. Usually, there is a positive dose–response relationships between exercise duration/frequency and depressive symptoms improvement [103]. In depressed rats, a single 30-min session of wheel running reduced the serum corticosterone concentration compared to 20 min [104]. In addition, 8-week high-frequency (3–5 sessions/week) aerobic exercise significantly increased serotonin and amygdala norepinephrine in the hippocampus of the brain of patients with depression compared to low-frequency (1 session/week) aerobic exercise [105]. Moreover, high-frequency exercise accelerated the serum BDNF peak, which promoted adaptation of central neurotransmitter release and was more effective at reducing depressive symptoms [106]. For adolescents, the shorter the effective time of physical exercise, the easier it is to improve the motivation of the adolescents to participate in physical exercise [107]. Reduced energy level is a characteristic symptom in depressed patients, and long-term continuous exercise may be too demanding for them. Furthermore, a meta-analysis on exercise durations/frequencies showed that exercise that lasted ≤ 45 min/session reduced depression symptoms more than > 45 min/session, and ≥ 4 sessions/week had a greater effect than 2–3 sessions/week [108].

In addition, there are a total of 6 comparisons in this article detailing the control of exercise intensity in the experiment. For adolescents with depression, moderate intensity and self-selected intensity may be better exercise options for them. Since only one comparison of each level of intensity was identified, further experiments are needed to strengthen any specific conclusions that can be drawn. For adolescents with depressive symptoms, a subgroup analysis found that moderate intensity and high intensity have a good effect on reducing their depressive symptoms. Previous research showed that moderate- and high-intensity exercise has a stronger effect on depression than low-intensity exercise [109]. The American Sports Medicine Association recommends 60–80% HRmax intensity to treat depression [110]. Exercise intensity is positively associated with BDNF and plasma endorphin release [111]. However, one study found that compared to high-intensity interval training, moderate-intensity continuous aerobic exercise reduced the levels of inflammatory factors such as TNF-α, IL-6, and IL-1β [112]. Given the low self-esteem and self-efficacy of depressed patients, moderate-intensity exercise is currently used more frequently [113]. Compared to self-selected intensity, exercise at a prescribed intensity usually leads to a poorer emotional experience [114]. The single included article on self-selected exercise intensity among adolescents with severe depression found that there was no significant difference in depression scores between the exercise and control groups after 6 weeks of intervention, but the exercise group had a significantly higher improvement at the 6-month follow-up. Further studies to examine the effectiveness of self-selected physical activity intensity on depression in adolescents are needed. As the suitable exercise intensity differs between adolescents and adults, it is necessary to further explore the effect and acceptability of different intensities on depression in adolescents in order to find the optimum intensity.

This meta-analysis has several limitations. (1) Only published Chinese and English articles were included, so the comprehensiveness of the search was limited. (2) Only two of the included articles fully described allocation concealment and only one mentioned blinded outcome assessment. (3) All the articles used subjective self-reported outcomes, with a lack of objective evaluation (such as biomarkers). (4) There was a high degree of heterogeneity among the studies of adolescents with depressive symptoms. (5) There was a lack of uniform standards and controls for exercise intensity variables. (6) The physical activity level of the subjects may affect the intervention effect, while this variable was not found or included when compiling relevant data. (7) The optimal exercise program in this research was based on the summary of current evidence. More RCTs are needed in the future to further discuss the intervention effects of different variables.

## Conclusion

This study shows that physical exercise, as an alternative or complementary treatment, has a positive effect on alleviating depression in adolescents, with a moderate effect size. Based on the current evidence, for adolescents with depression lasting for 6 weeks, a physical exercise program of 30 min/time, 4 times/week, and aerobic exercise is better. For adolescents with depressive symptoms lasting for 8 weeks, 75–120 min/time of exercise 3 times/week, and aerobic exercise is better. Physical exercise of moderate intensity is a better choice for adolescents with depression and depressive symptoms. In the future, empirical research should involve long-term, high-quality RCTs, and increased follow-up to explore the sustained benefits of physical exercise. The forms of exercise intervention among adolescents should be further enriched, and the control of the intensity of physical exercise should be strengthened as well.

## Data Availability

The datasets used and/or analyzed during the current study are available from the corresponding author on reasonable request.
